# Bio-bleaching of Remazol brilliant blue-19 by *Stereum ostrea*

**DOI:** 10.1007/s13205-015-0301-x

**Published:** 2015-05-07

**Authors:** K. Praveen, K. Y. Usha, Kanderi Dileep Kumar, Sake Pradeep, B. Rajasekhar Reddy

**Affiliations:** Department of Microbiology, Sri Krishnadevaraya University, Anantapuramu, Andhra Pradesh India; Department of Microbiology, Yogi Vemana University, Kadapa, Andhra Pradesh India

**Keywords:** Remazol brilliant blue-19, *S. ostrea*, *P. chrysosporium*, Optimization, Lignolytic enzymes, Dye effluent

## Abstract

Efficiency of white-rot fungi—*Stereum ostrea* (*S. ostrea*) as a test culture and *Phanerochaete chrysosporium* (*P. chrysosporium*) as a reference culture in colour removal from a textile dye, Remazol brilliant blue-19 (RBB-19) in medium was compared in this study. *S. ostrea* was more efficient than *P. chrysosporium* in decoloration process. Different parameters pH, temperature, sources of carbon and nitrogen, stationary and shaking conditions were optimized for bleaching of dye by the fungal cultures. Optimal growth conditions for decoloration of dye by both cultures were pH 5.0, temperature 35 °C, glucose and fructose as best carbon source at 1 % level, peptone and urea as best nitrogen source and shaking conditions (150 rpm). Culture broth free of colour (99 % of decoloration) was achieved with *S. ostrea* as against 70 % decoloration by *P. chrysosporium* on 6th day of incubation. Adsorption of dye to fungal biomass as reflected by colour coating on biomass and participation of lignolytic enzymes in colour removal appeared to be mechanisms involved in decoloration process. The ability of both fungal cultures in removal of colour in effluents with dyes collected from silk saree-weaving cottage unit was tested. *S. ostrea* was also found to be more effective in colour removal from effluent. *S. ostrea* appears to be a promising culture for application of bioremediation in decoloration of dyes.

## Introduction

Synthetic dyes are a group of organic aromatic molecular structural compounds that are extensively used in textile, paper, printing and dye houses (Lilly and Barnett 1951; Jacob and Azariah 2000; Aksu 2005) . According to their dissociation in an aqueous solution, dyes can be classified as acid, direct reactive dyes (anionic), basic dyes (cationic) and disperse dyes (nonionic) (Mishra and Tripathi 1983). Many chemical dyes are being used increasingly in textile and dyeing industries because of their ease and cost effectiveness in synthesis, firmness and variety in colour compared to that of natural dyes (Mathur et al. 2005). India’s dye industry produces every type of dyes and pigments. Production of dyestuff and pigments in India is close to 80,000 tonnes per annum (Mathur et al. 2005). India is the second largest exporter of dyestuffs and intermediates after China (Mathur et al. 2005).

The textile industry accounts for the largest consumption of dyestuffs, at nearly 80 % (Mathur et al. 2005). According to the survey of the Ecological and Toxicological Association of the Dyestuffs Manufacturing Industry (ETAD), over 90 % of some 4000 dyes have LD_50_ values greater than 2000 mg/kg (Robinson et al. 2001) which is very dangerous to nature. Effluents from the textile industries containing dyes are highly coloured and are, therefore, visually identifiable (Kilic et al. 2007). The coloured effluents damage the aesthetic quality of water and soil and reduce light penetration and photosynthesis and also some of the dyes are toxic or mutagenic, carcinogenic and allergenic (Kumar et al. 2006). Because of the widespread use and potential carcinogenicity of certain dyes, there has been a growing interest in assessing the hazards associated with dyes available in local markets and hence decoloration of the dye-bearing effluents is of great importance. Everything touched by king Midas turned to gold. By a sort of inversion process, everything that modern men touch turns to a waste product sooner or later. Wastes are usually discarded into water, with or without processing. At present, water is becoming a rare commodity, and the available water resources are inadequate to meet the essential needs of man. Improper disposal methods and inadequate control of toxic effluents from different industries have led to the widespread contamination of surface as well as groundwater and have made the water resources unfit for usage (Odum et al. 1969).

The traditional physical or chemical decoloration methods including coagulation, flocculation, ion exchange, irradiation, precipitation, ozonation and adsorption or a combination of these methods have been used for dye removal from wastewaters (Akar et al. 2009). However, application of these methods is somewhat restricted due to some limitations such as operational costs, formation of hazardous by-products, intensive energy requirement (Padmesh et al. 2005) and limited adaptability to a wide range of effluents (Fu and Viraraghavan 2001). Dye removal from waste water by established waste water treatment processes are expensive and need careful application (Vandevivere et al. 1998). Furthermore, following anaerobic digestion, nitrogen-containing dyes are transformed into aromatic amines that are more toxic and mutagenic than the parent molecules (Ganesh et al. 1999). To overcome these difficulties, microorganisms are being investigated for their potential to bleach dye-bearing effluents. Biotechnological approaches were proven to be potentially effective in treatment of this pollution source in an eco-efficient manner (Robinson et al. 2001). Although many microorganisms belonging to different taxonomic groups of bacteria (Wu et al. 2005) and algae (Dilek et al. 1999) have been reported for their ability to bleach different dyes, fungi, in particular, white-rot group are recognized for their superior capacity to produce extracellular lignolytic enzymes such as laccase (Lac), lignin peroxidase (LiP) and manganese peroxidase (MnP) (Gao et al. 2010; Praveen et al. 2011) which are highly oxidative and nonspecific in action and responsible for degradation and decoloration of a wide range of dyes (Aksu 2005; Praveen et al. 2011). The capacity of organisms in bio-bleaching process of dye differs from one organism to another and is dependent on inherent capacity of the organism, growth conditions and structure of dye. This is quite evident from a comparative study (Baldrian and Snajdr 2006) that five litter-decomposing fungi (LDF) and two reference white-rot cultures exhibited difference in rates of bio-bleaching of 4 dyes including RBB-19 in high nitrogen and high carbon (HNHC) medium. According to this study, the reference culture *Trametes versicolor * and LDF *Collybia dryophilia* displayed the fastest degradation of the dye Poly B-411 whereas other cultures LDF X1 and X2 caused most rapid decoloration of RBB-19. Similarly, *Stropharia rugosoannulata* was found to decolorise efficiently anthraquinone dye Basic Blue 22 (Jarosz-Wilkolazka et al. 2002). In this study, we report the ability of *S. ostrea* to bleach the dye—RBB-19 in medium and effluents from silk saree-weaving cottage unit in comparison to the reference culture *P. chrysosporium* whose potential in color removal of dyes was assessed earlier (Rojek et al. 2004).

## Materials and methods

### Fungal cultures

The fungal cultures—*S. ostrea* and *P. chrysosporium*—were maintained on Koroljova-skorobogat’ko medium (1998) containing the following composition (g/l): 3.0 peptone, 5.0 glucose, 0.6 KH_2_PO_4,_ 0.5 MgSO_4, _0.4 K_2_HPO_4, _0.05 MnSO_4, _0.001ZnSO_4,_ 0.0005 FeSO_4_, 20.0 agar (pH 6.0).

### Dye characterization

**Fig. 1:**
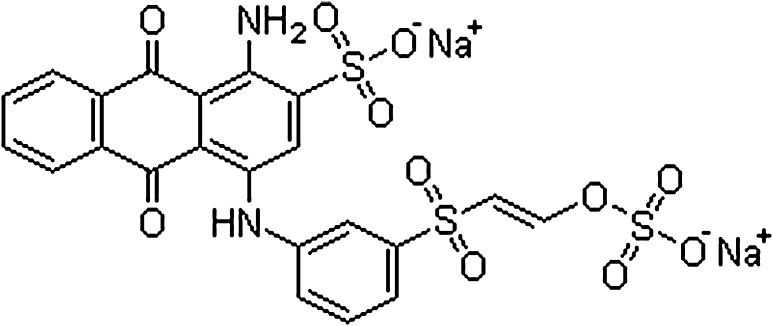
Chemical structure of RBB-19

RBB-19 is one of the dyes used for colouring textile fibers and was provided by a dyeing unit, Dharmavaram, Anantapuramu district of Andhra Pradesh, India. Chemical structure of the selected dye is specified in Fig. 1. Spectrophotometric scanning of dye solution was performed in a UV–Visible spectrophotometer (Chemito-UV-2600) and the absorption maximum of RBB-19 was identified as 590 nm.

### Growth conditions for bleaching

All experiments with both fungal cultures were conducted in Koroljova liquid medium in 250-ml Erlenmeyer flasks. Fungal mycelial suspension was prepared by adding 2 ml of sterile distilled water to the freshly grown 7-day slants of the above cultures. The homogenized mycelial suspension of cultures was used to inoculate 50 ml of liquid media amended with RBB-19 at 0.02 % concentration (unless specified) into 250-ml Erlenmeyer flasks and all flasks were incubated for growth at 30 °C and speed of 200 rpm in an incubator cum shaker (ORBITEK, India).

Triplicate flasks were withdrawn daily and harvested by passing through Whatman filter paper No.1 for collection of supernatant and fungal biomass. Supernatant/culture filtrate, free of fungal biomass, was spun at 10,000*g* for 15 min in a cooling centrifuge (REMI C24-BL) and used for estimation of bleaching. Dry weight of fungal biomass on filter paper after drying in an oven at 70 °C was quantified.

### Optimization for bleaching of dye

For optimization of bleaching of dye RBB-19, only one of the factors of physical parameters and nutrient parameters in growing medium was varied at a time by keeping all other factors constant. Both cultures were grown in presence of dye RBB-19 at 0.02 % concentration and bleaching experiments were repeated in the same manner as mentioned above. For determination of optimal pH, Koroljova medium after amendment of dye was adjusted to initial desired pH within a range of 3.0–7.0. Cultures were grown on medium set to desired pH and bleaching of dye was estimated in the same way as done earlier. For proceeding to optimize the next factor, Koroljova medium with dye was set to optimal value of pH 6.0 and incubated at different temperatures (20–50 °C) after inoculation of fungal cultures in an orbital shaker at speed of 200 rpm. The extent of bleaching that took place was estimated in the same manner as described previously. For selection of the best carbon source, sucrose, fructose, maltose or starch at 1 % concentration was included in Koroljova medium amended with dye RBB-19 at 0.02 %. Cultures were grown in the same manner as described earlier. The percentage of decoloration of dye was recorded. Experiment was repeated in the same way as carbon source for selection of the best nitrogen source. For this purpose peptone of Koroljova medium was replaced with different nitrogen sources yeast extract, ammonium sulphate, ammonium nitrate or urea at 1 % level.

### Effect of stationary and shaking conditions

To determine the effect of stationary and shaking conditions on decoloration of RBB-19 by the fungal cultures, one set of flasks with medium containing dye was incubated at 30 °C under stationary conditions. Four sets of flasks with medium with cultures were incubated in a rotary shaker at speed of 50, 100, 150 and 200 rpm at 30 °C for 6 days. Flasks were processed on 6th day of incubation for assessment of decoloration in the same manner as described earlier.

### Effect of initial dyestuff concentrations

To test the capacity of both cultures to bleach the highest concentration of RBB-19, dye was included in optimized medium at varying concentrations within a range 0.04–0.2 %. Cultures were grown in the medium under optimal conditions and processed for assessment of bleaching in the same manner as done for previous experiments.

### Assay of lignolytic enzymes

Activities of lignolytic enzymes in the cultural filtrate of both fungal cultures derived from growth on Koroljova medium with dye were estimated following the standard protocols. Laccase activity was assayed using 10 mM guaiacol in 100 mM acetate buffer (pH 5.0) containing 10 % (V/V) acetone. The change in absorbance of the reaction mixture containing guaiacol was monitored at 470 nm (*ε* = 6740 M^−1^ cm^−1^) for 5 min of incubation (Das et al. 1997). Laccase activity was expressed in International Units (IU) where one unit corresponded to the amount of enzyme that oxidized one micromole of guaiacol per minute. Lignin peroxidase activity was determined by oxidation of veratryl alcohol at 310 nm (*ε* = 9300 M^−1^ cm^−1^) (Tien and Kirk  1988). The reaction mixture was composed of 0.5 ml culture filtrate, 0.4 mM H_2_O_2_ and 50 mM tartaric acid (pH 2.5) and 2 mM veratryl alcohol. The enzyme activity was expressed in IU where one unit of LiP corresponded to the amount of enzyme that oxidized one micromole of veratryl alcohol per min. MnP activity was determined by oxidation of phenol red at 610 nm (Kuwahara et al. 1984). The assay mixture included 0.5 ml culture filtrate, 0.25 M sodium lactate (pH 4.5), 0.5 % bovine albumin, 200 mM MnSO_4_, 2.0 mM H_2_O_2_ (prepared in 0.2 mM sodium succinate buffer pH 4.5) and 0.1 % phenol red. The change in absorbance of reaction mixture was monitored at 610 nm (*ε* = 22,000 M^−1^ cm^−1^) for 5 min. MnP activity was expressed in IU where one unit of MnP was defined as the amount of enzyme that oxidized one micromole of phenol red per min.

### Bleaching of effluent

Effluent was collected from a local dyeing unit of silk sarees, Dharmavaram, Andhra Pradesh, India. The effluent was checked for pH which was found to be 11.0. Scanning of the effluent in a UV–Visible spectrophotometer (Chemito-UV-2600) indicated the presence of dye RBB-19 in effluent. After adjustment of pH 5.0, effluent was amended with glucose at 1 and 2 % concentration and was grown with fungal cultures under optimal conditions in 250-ml Erlenmeyer conical flasks in the same manner as specified above. The flasks were processed for assessment of bleaching of dye on 6th day of incubation.

### Decoloration assay

At the end of each experiment the supernatants were subjected to dye decoloration analysis. Medium without dye and inoculum and dye-amended medium without inoculum were maintained as controls. Absorbance of colour of dye in the uninoculated medium amended with dye was measured against uninoculated medium without dye at 590 nm at any given time interval and is treated as absorbance of control. Absorbance of colour of dye in the culture filtrate derived from the growth of fungi was measured against uninoculated medium without dye at 590 nm at the respective time interval and was considered as observed absorbance. Decoloration was expressed as activity (%).

$${\text{Decolorization \% }} = \frac{{{\text{Control absorbance}} - {\text{Observed absorbance}}}}{\text{Control absorbance}}\; \times \; 100$$

## Results

Knowledge of nutrient requirements and conditions for growth of unexplored organisms is essential for their effective utilization in bio-bleaching of dyes. Hence, experiments were conducted with *S. ostrea* to widen our knowledge base.

### Decolorization of RBB-19 by fungal cultures

**Fig. 2:**
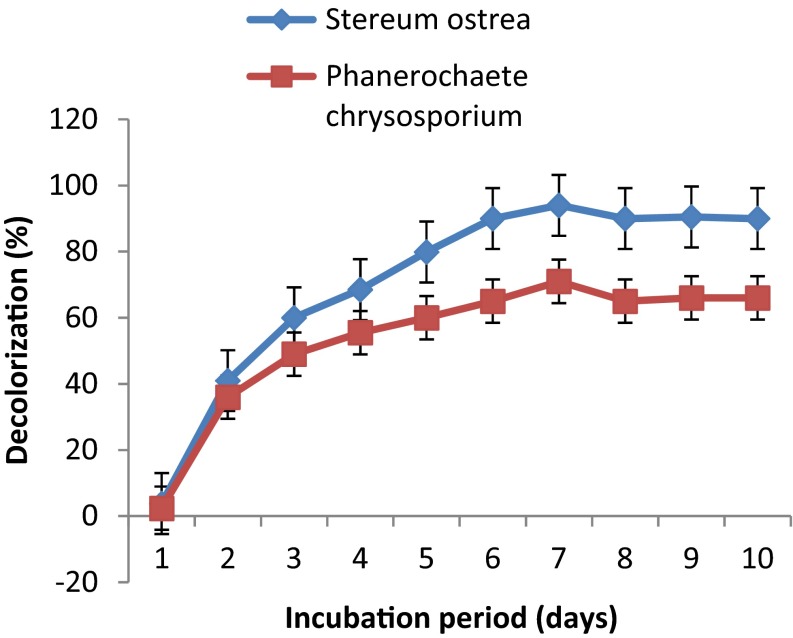
Decoloration of RBB-19 by fungal cultures

Growth of the fungal cultures in defined Koroljova medium in the presence of dye, RBB-19 at 0.02 % for 10 days resulted in bleaching of dye (Fig. 2). Dye decoloration was observed from the first day onwards in both cultures. Maximum decoloration of dye by *S. ostrea* cultures was found to be 93 % on 6th day of incubation whereas *P. chrysosporium* could achieve only 65.5 %. Further incubation of the cultures did not improve colour removal.

### Effect of initial pH and temperature on decoloration

**Fig. 3:**
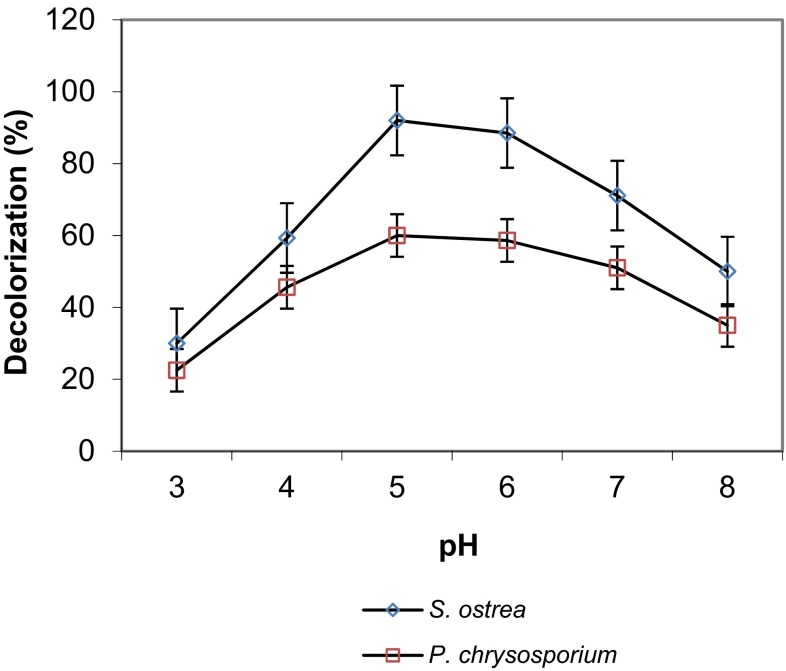
Effect of pH on decoloration of RBB-19 by fungal cultures on 6th day of incubation

Decoloration of RBB-19 in cultures of *S. ostrea* and *P. chrysosporium* grown on Koroljova medium at different pH levels (3.0–8.0) is presented in Fig. 3. Initial pH of medium for growth of the cultures had influence on decoloration of dye. The decoloration of dye by these cultures was found to be optimal at pH 5.0. *S. ostrea* caused 94.5 % decoloration of dye as against 63.9 % by *P. chrysosporium* at pH 5.0 on 6th day of incubation. There was a drop in bleaching of dye by the fungal cultures on either side of the optimal pH.

**Fig. 4:**
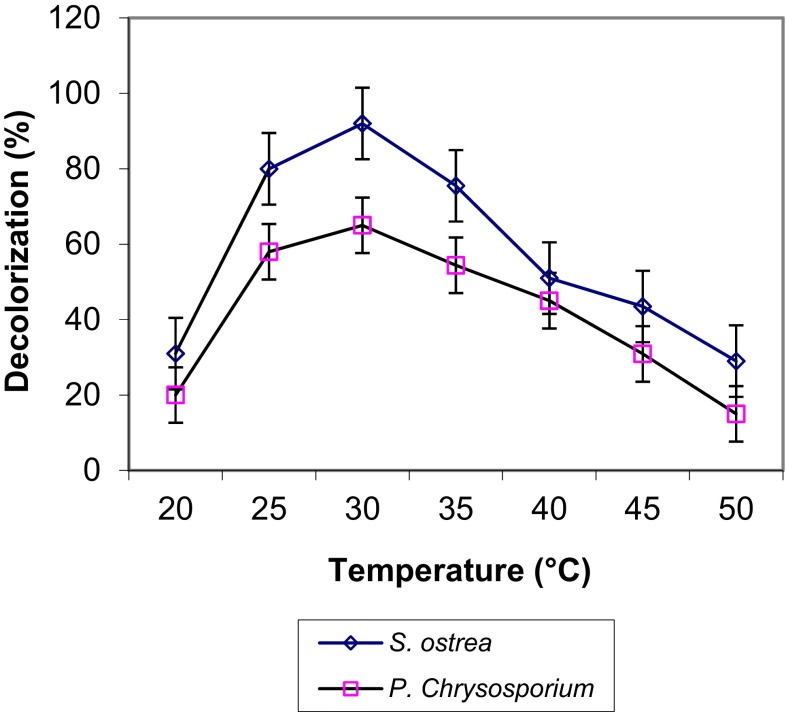
Effect of temperature on decoloration of RBB-19 by fungal cultures on 6th day of incubation

The influence of various temperatures (20–60 °C) on decoloration of RBB-19 is shown in Fig. 4. Maximum decolorization of 95.7 and 65 % occurred in cultures of *S. ostrea* and *P. chrysosporium* grown at 35 °C, respectively. The decreasing trend of decoloration was noticed in cultures grown at higher temperatures (50–60 °C).

### Effect of concentration of carbon sources (1 %) on decoloration

**Fig. 5:**
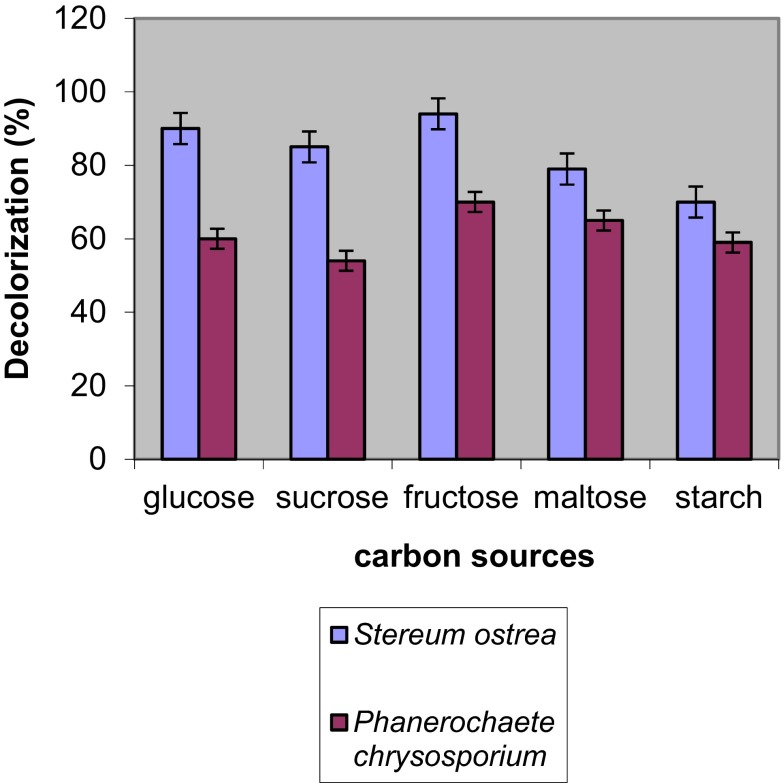
Effect of carbon sources on decoloration of RBB-19 by fungal cultures on 6th day of incubation

Different carbon sources were added into medium to study their effect on decoloration efficiency by fungal cultures (Fig. 5). Maximum colour removal of 98.5 and 68 % by *S. ostrea* and *P. chrysosporium* was achieved with glucose and fructose followed by sucrose on 6th day of incubation, respectively.

### Effect of nitrogen sources on decoloration

**Fig. 6:**
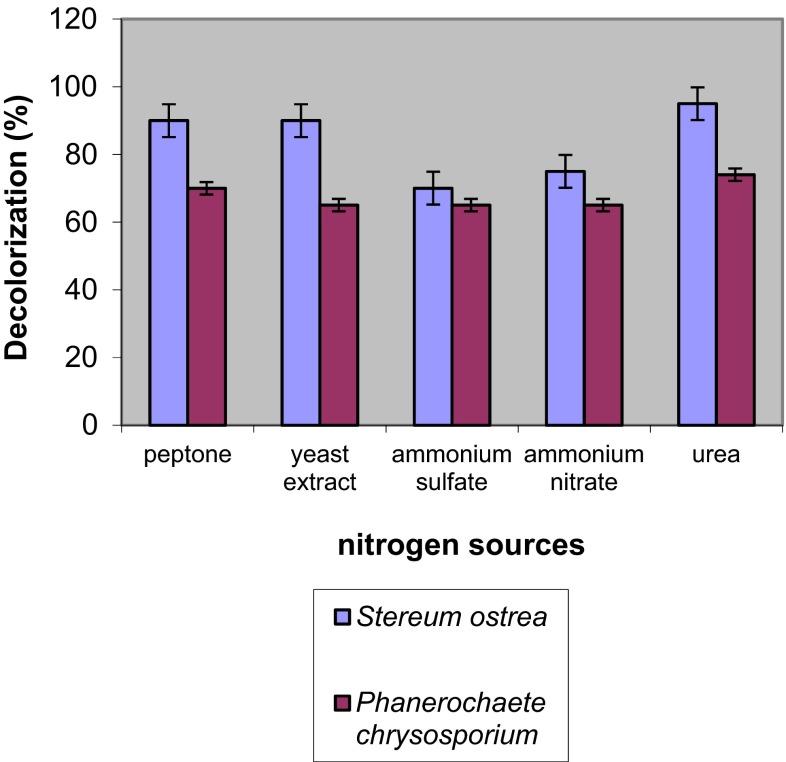
Effect of nitrogen sources on decoloration of RBB-19 by fungal cultures on 6th day of incubation

Bleaching of dye in cultures grown on medium with various nitrogen sources on 6th day of incubation was assessed by *S. ostrea* and *P. chrysosporium* (Fig. 6). Maximum decoloration of dye to the tune of 99 and 70 % was achieved on medium with urea followed by peptone on 6th day of incubation. However, the percent of decoloration on medium containing other nitrogen sources was lower.

### Effect of stationary and shaking conditions

Decoloration of RBB-19 in the fungal cultures grown under stationary and shaking conditions with different speed on 6th day of incubation was examined (Table 1). Both fungal cultures exhibited maximum percent of decoloration under shaking conditions as compared under stationary conditions. *S. ostrea* and *P. chrysosporium* could bring out 99 and 80 % of decoloration under shaking conditions at 150 rpm as against 90.8 and 58 % under stationary conditions, respectively (Table 1). Although agitation/shaking at 150 rpm was more beneficial to *P. chrysosporium* in bleaching dye, *S. ostrea* performed better than *P. chrysosporium* on bleaching of dye.

**Table 1:** Effect of stationary and shaking on decoloration of RBB-19 by fungal cultures on 6th day of incubation

Fungus	% Decolorization of dye in the culture filtrate				
	Stationary conditions	Shaking conditions			
		50 rpm	100 rpm	150 rpm	200 rpm
*S. ostrea*	90.8	94.5	97.9	99	99
*P. chrysosporium*	58	62.5	75.8	80	80

### Effect of dye concentration on decoloration

**Fig. 7:**
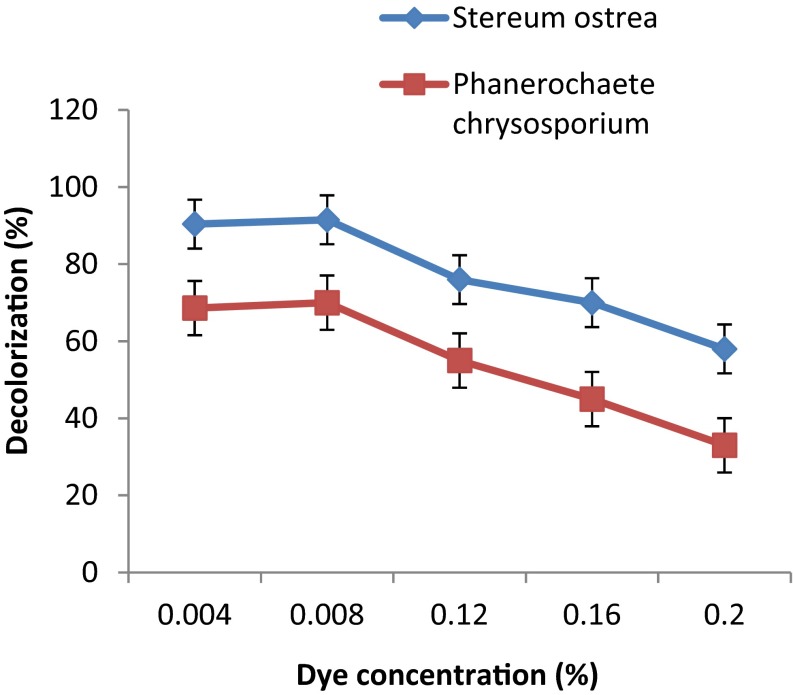
Effect of dye concentration on decoloration of RBB-19 by fungal cultures on 6th day of incubation

The concentration of dye, RBB-19, in the medium had influence on decoloration of dye by both fungal cultures (Fig. 7). The rate of decoloration was highest at the lowest concentration of dye (0.04 %) used in the medium as reflected by high percentage of decoloration by *S. ostrea* (96.7) and *P. chrysosporium* (71.5). The percent of decoloration gradually decreased as the concentration of dye in the medium was increased from 0.04 to 0.20 %.

### Lignolytic enzymes of the fungal cultures

Growth of both cultures on optimized medium in the presence of dye, RBB-19, at 0.02 % concentration under optimal conditions exhibited three extracellular lignolytic enzymes Lac, LiP and MnP. Yields of Lac to the tune of 51 U/ml by *S. ostrea* on 6th day of incubation was recorded as against only 8.5 U/ml by *P. chrysosporium*. Titers of LiP and MnP in respect of *S. ostrea* were 1.9 and 3.1 U/ml, respectively, whereas the corresponding figures of enzymes in respect of *P. chrysosporium* were 0.52 and 1.07 U/ml.

### Effluent treatment

Effluent carrying RBB-19 was amended with glucose and was set to pH 5.0. The performance of both fungal cultures on decoloration of effluent was tested. The fungal cultures *S. ostrea* and *P. chrysosporium* could bleach dye to the tune of 70 and 43 % in effluent amended with 1 % glucose on 6th day of incubation, respectively. Increase of glucose to 2 % in effluents was not beneficial to decoloration. The percentage of decoloration of dye in respect of *S. ostrea* and *P. chrysosporium* recorded on 6th day of incubation was 65 and 40 %, respectively.

## Discussion

The performance of white-rot basidiomycetes *S. ostrea* and *P. chrysosporium* on decoloration of dye, RBB-19, at 0.02 % concentration in defined medium Koroljova medium under controlled conditions in shake flask for 10 days was examined in this study. *S. ostrea* was more efficient in colour removal than *P. chrysosporium* under growth conditions employed in this study.

Though *S. ostrea* exhibited bleaching of dye to a greater extent, maximum decoloration occurred in both cultures grown on medium set to pH 5 in the present study. At high pH the growth of both cultures was poor and even decoloration of dye was slow. Growth (data not shown) and decoloration proceeded in parallel at different pH. This is consistent with result of different white-rot fungi in a majority of studies (Kapdan et al. 2000; Asghar et al. 2006; Pallavi et al. 2011) that maximum growth of organisms and decoloration took place in acidic pH range. Decoloration of RBB-19 by both fungal cultures in the present study at different temperatures showed 35 °C as an optimum temperature. This is consistent with temperature optima of 30–37 °C for decoloration mediated by mesophilic white-rot fungi (Renganathan et al. 2006; Pallavi et al. 2011).

Both cultures, *S. ostrea* and *P. chrysosporium*, exhibited the same optimal conditions for decoloration of the dye, RBB-19 in the present study. The optimal conditions for bio-bleaching of the dye by the same cultures were pH 5.0, temperature 35 °C, and glucose and fructose as the best carbon and peptone and urea as the best nitrogen source. The best decoloration efficiencies (65–80 %) of the dye were obtained when *Trametes versicolor* grown in nitrogen-limited conditions under aerobic conditions in a fed-batch study (Moreira et al. 2004). *Trametes pubescens* completely removed colour of two anthraquinone dyes RBB-19 and B-49 in low-nitrogen minimal medium (LNMM) in bioreactor for 5 cycles with increasing concentration up to 1000 ppm.

Shaking was beneficial to decoloration of dye by the fungal cultures in particular to *P. chrysosporium* in the present study. Similar observations on decoloration of different dyes by a number of organisms, *P. chrysosporium, Trametes* spp, were made (Glenn et al. 1983; Kumar et al. 1998).

The rate of decoloration of dye by both fungal cultures in the present study decreased with increase in the concentration of dye from 0.04 to 0.2 % in the medium. Growth of the both fungal cultures at high concentration used in the study was severely affected. An observation of inverse relationship between concentration of dye in the medium and decoloration is in agreement with results of other study (Jarosz-Wilkolazka et al. 2002) that higher concentration (600–1000 mg/L) of reactive blue in the medium adversely affected both adsorption to mycelia and decoloration by *Aspergillus ochraceus* NCIM-1146. Fall in decolorizing activity of the fungal cultures at higher concentration of dye could be due to exhaustion of limited binding sites on biomass for adsorption even at lower concentration of dye.

Bleaching of color that appeared on mycelial mat of the cultures in the present study is probably due to involvement of lignolytic enzymes. Growth of *S. ostrea* in the presence of RBB-19 produced higher biomass (data not shown) and higher yields of lignolytic enzymes Lac, LiP and MnP than that of *P. chrysosporium* under conditions employed in the present study. In view of participation of lignolytic enzymes in decoloration, the presence of lignolytic enzymes in fungal cultures is routinely tested on decoloration assay with Poly 478 dye (Pointing 1999). Bleaching of RBB dye at high rate by *S. ostrea* in the present study could be attributed to its higher growth and its larger capacity to secrete lignolytic enzymes in higher titres. Evaluation of performance of the two cultures in shake flasks in removal of color from coloured waste water collected from a local cottage dying unit indicated that *S. ostrea* was more effective than *P. chrysosporium*.

Addition of 1 % glucose to waste water enhanced decoloration process whereas, increase in concentration of glucose results in decrease in decoloration. A similar observation was made that *P. chrysosporium* and *Coriolus versicolor* required only additional labile carbon–glucose in decoloration of anaerobically digested molasses spent wash (Kumar et al. 1998).

In summary, the isolate *S. ostrea* is better than the reference culture *P. chrysosporium* for decolorization of reactive textile dye. Even though both cultures displayed the same and identical optimal conditions, *S. ostrea* secreted lignolytic enzymes in larger amounts. *S. ostrea* performed better than *P. chrysosporium* in removal of color from effluent.

## References

[CR1] Akar ST, Akar T, Cabuk A (2009). Decolourization of a textile dye, reactive red 198 (RR198), by *Aspergillus parasiticus* fungal biosorbent. Braz. J Chem Eng.

[CR2] Aksu Z (2005). Application of biosorption for the removal of organic pollutants: a review. Process Biochem.

[CR3] Asghar M, Asad MJ, Legge RL (2006). Enhanced lignin peroxidase synthesis by *Phanerochaete chrysosporium* in solid state bioprocessing of a lignocellulosic substrate. World J Microbiol Biotechnol.

[CR4] Baldrian P, Snajdr J (2006). Production of lignolytic enzymes by litter-decomposing fungi and their ability to decolorize synthetic dyes. Enz Microbiol Technol.

[CR5] Das N, Sengupta S, Mukherjee M (1997). Importance of Laccase in vegetative growth of *Pleurotus florida*. Appl Environ Microbiol.

[CR6] Dilek FB, Taplamacioglu HM, Tarlan E (1999). Color and AOX removal from pulping effluents by algae. Appl Microbiol Biotechnol.

[CR7] Fu Y, Viraraghavan T (2001). Fungal decolourization of dye wastewaters: a review. Bioresour Technol.

[CR8] Ganesh R, Boardman GD, Michelsen D (1999). Fate of azo dyes in sludge’s. Water Res.

[CR9] Gao D, Du L, Yang J, Wu WM, Liang H (2010). A critical review of the application of white rot fungus to environmental pollution control. Critical Rev Biotechnol.

[CR10] Glenn JK, Morgan MA, Mayfield MB, Kuwahara M, Gold MH (1983). An extracellular H_2_O_2_-requiring enzyme preparation involved in lignin biodegradation by the white rot basidiomycete *Phanerochaete chrysosporium*. Biochem Biophys Res Commun.

[CR11] Jacob CT, Azariah J (2000). Environmental ethical cost of t-shirts, Tiruppur.

[CR12] Jarosz-Wilkolazka A, Kochmanska-Rdest J, Malarczyk E, Wardas W, Leonowicz A (2002). Fungi and their ability to decolourize azo and anthraquinonic dyes. Enz Microbiol Technol.

[CR13] Jarosz-Wilkolazka A, Kochmanska-Rdest J, Malarczyk E, Wardas W, Leonowicz A (2002). Fungi and their ability to decolourize azo and anthraquinonic dyes. Enz Microbiol Technol.

[CR14] Kapdan IK, Kargi F, McMullan G, Marchant R (2000). Effect of environmental conditions on biological decolorization of textile dyestuff by *C. versicolor*. Enz Microbiol Technol.

[CR15] Kilic NK, Nielson JP, Yuce M, Donmez G (2007). Characterization of a simple bacterial consortium for effective treatment of wastewaters with reactive dyes and Cr(VI). Chemosphere.

[CR16] Koroljova-Skorobogat’Ko OV, Stepanova EV, Gavrilova VP, Morozovaa OV, Lubimova NV, Dzchafarova AN, Jaropolov AI, Makower A (1998). Purification and characterization of the constitutive form of laccase from the basidiomycetes *Coriolushirsutus* and effect of inducers on laccase synthesis. Biotechnol Appl Biochem.

[CR17] Kumar V, Wati L, Nigam P, Banat IM, Yadav BS, Singh D, Marchan R (1998). Decolorization and biodegradation of anaerobically digested sugarcane molasses spent wash effluent from biomethanation plants by white-rot fungi. Proc Biochem.

[CR18] Kumar KV, Ramamurthi V, Sivanesan S (2006). Biosorption of malachite green, a cationic dye onto *Pithophora* sp., a fresh water algae. Dyes Pigment.

[CR19] Kuwahara M, Glenn JK, Morgan MA, Gold MH (1984). Separation and characterization of two extracellular H_2_O_2_-dependent oxidases from lignolytic cultures of *Phanerochaete chrysosporium*. FEBS Lett.

[CR20] Lilly VM, Barnett HL (1951). Physiology of the fungi.

[CR21] Mathur N, Bhatnagar P, Bakre P (2005). Assessing mutagenicity of textile dyes from Pali (Rajasthan) using Ames bioassay. Appl Eco Environ Res.

[CR22] Mishra G, Tripathi M (1983). A critical review of the treatments for decolourization of textile effluent. Colourage.

[CR23] Moreira MT, Viacava C, Vidal G (2004). Fed-batch decolorization of Poly R-478 by *Trametes versicolor*. Braz Arch BiololTechnol.

[CR24] Odum EP (1969) In: Fungal physiology, 2nd edn. Saunders, Philadelphia

[CR25] Padmesh TVN, Vijayaraghavan K, Sekaran G, Velan M (2005). Batch and column studies on biosorption of acid dyes on fresh water macro algae *Azollafiliculoides*. J Hazard Mater.

[CR26] Pallavi H, Viswanath B, Rajasekhar Reddy B. Decolorisation of dyes by fungi 2011. Lambert Academic Publications (LAP). ISBN 13: 9783846530344ISBN 10: 3846530344, p 152

[CR27] Pointing SB (1999). Qualitative methods for the determination of lignocellulolytic enzyme production by tropical fungi. Fungal Divers.

[CR28] Praveen K, Viswanath B, Usha KY, Pallavi H, Reddy GVS, Naveen M, Reddy BR (2011). Lignolytic enzymes of a mushroom—*Stereumostrea* isolated from wood logs. Enz Res.

[CR29] Renganathan S, Thilagaraj WR, Miranda LR, Gautam P, Velan M (2006). Accumulation of acid orange 7, acid red 18 and reactive black 5 by growing *Schizophyllum commune*. Bioresour Technol.

[CR30] Robinson T, McMullan G, Marchant R, Nigam P (2001). Remediation of dyes in textile effluent: a critical review on current treatment technologies with a proposed alternative. Bioresour Technol.

[CR31] Rojek K, Roddick FA, Parkinson A (2004). Decolorization of natural organic matter by *Phanerochaete chrysosporium*: the effect of environmental conditions. Water Sci Technol Water Suppl.

[CR32] Tien M, Kirk TK, Wood WA, Kellogg ST (1989). Methods in Enzymology: Biomass, part b. Lignin, Pectin and Chitin.

[CR33] Vandevivere PC, Bianchi R, Verstraete W (1998). Treatment and reuse of wastewater from the textile wet-processing industry: review of emerging technologies. J Chem Technol Biotechnol.

[CR34] Wu JY, Hwang SCJ, Chen CT, Chen KC (2005). Decolorization of azo dye in a FBR reactor using immobilized bacteria. Enz Micro Biol Tech.

